# Whose Art Are We Talking About?

**DOI:** 10.1007/s10699-015-9451-x

**Published:** 2015-10-29

**Authors:** Ike Kamphof

**Affiliations:** Faculty of Arts and Social Sciences, Maastricht University, Box 616, 6200 MD Maastricht, Netherlands

**Keywords:** Art of living, Telecare, Technomoral change, Reflexivity, Relational being

## Abstract

Jeannette Pols and Tamar Sharon kindly reviewed my case study of the art of living with technology as an engagement with technomoral change. I am indebted to them for their careful reading and critical suggestions to further elaborate the project. In my response I focus on the question whose art we are talking about, while further elucidating the reflexivity addressed in my essay. I conclude with some remarks on what we can learn from micro studies like the one presented for macro level thinking on the ‘art of becoming’ with technology in a surveillance society.

Pols strongly supports my focus on the aesthetic evaluation of the emerging network of relationships in technologically mediated homecare. She is too modest as I am leaning on her own work in this area, connecting it with earlier studies of professional artistry as reflection-in-action and with recent work on processes of technomoral change. Pols emphasizes that an aesthetic approach needs to consider all partners in the network, where I have focused on caregivers as moral actors in their relationship with their clients. She specifically suggests, partly referring to my own work (Kamphof 2013), to address the form of the technology discussed, that forces caregivers to repair tensions in the relationship with their clients they haven’t asked for. Furthermore, Pols argues for technologies that are aesthetically pleasing to their users and for more space for experiment in new technologically mediated care practices. Responding to these recommendations, I will clarify some points about the case discussed and subsequently address the more general question at stake here: whose art of living—or lack of such an art—are we talking about?

Monitoring technologies allegedly enable frail elderly people to live in their own homes longer by catching emerging health and safety problems at an early stage. In order to bring about these potential benefits, homecare clients have to approve the installation of a system that registers data about their daily patterns of living far beyond what caregivers used to know about them. Not surprisingly, clients are ambiguous about this. On the one hand, as Pols suggests, many fear being left without recourse to help in case of accidents. On the other hand, many also resist being seen as the kind of person that needs continuous observation. They fear unsolicited intrusions into their lives. Clearly, the network of care under discussion does not only pose moral challenges to caregivers. Clients play an active role in ‘the art of relating’ that is required to make the new set-up work. They must allow the sensors to read their bodily behavior and have to develop trust in the system and in the caregivers monitoring their data. My fieldwork showed that mutual joking about the new technological possibilities often played a vital role in the relationship between clients and formal and informal caregivers observing them. Some clients indeed responded to the sensors and the imagined person behind them affectively, in one case by waving goodnight to the sensors, telling them they could stop now for the night.

The technological partner in the network could use improvement. By capturing data through small, easily forgotten motion sensors and transferring these data out of the home to be interpreted elsewhere, the system confirms ageist stereotypes of elderly people as incompetent. The message conveyed by the current form of the system to homecare clients is that their data are not their own, but belong to others who use them for taking decisions on care. These decisions may be oriented to the wellbeing of clients, but could also include paternalistic forms of control. At present, caregivers have to resolve the tensions that the system introduces into the relationship with their clients as a relationship of trust. They have to open conversations with their client about observations, who may have forgotten the system, and provide appropriate levels of transparency. With an adjusted design, the system could actively invite such conversations, for instance by providing a platform in the home where data are open to negotiation. Secondly, as Pols suggest, the sensors could be designed in such a way that they support feelings of being watched over in benign ways.

However, all of this can only succeed when the healthcare system at large supports the use of data primarily for the wellbeing of individual clients. In the case presented, management offered caregivers the freedom to experiment and take their own decisions. One can imagine alternative scenarios in which insurance companies and other commercial partners gain access to data and use them in ways that challenge relationships of trust on the work floor. In sum, securing trustworthy user practices requires the co-operation of many partners. Next to caregivers, clients and technologies, it should include engineers and designers developing technologies, and government agencies, insurance companies and healthcare managers financing and organizing the context of use. Representatives of these last groups would do well to safeguard space for experimentation. In their own way, they too have to be actively open to tensions technologies provoke and to the emerging meaning of technologies in practices of use, and respond to these with feeling and imagination.

Technological and human partners bring different capacities to the dance of ‘reflective implementation’ that would ideally result from such an active openness. Technologies do not feel their way about tautegorically the way humans do, nor do they act out of professional and personal commitment to challenged and cherished values. However, as indicated, they do embody values—fitting and unfitting ones. In their shape and materiality they carry (im)possibilities that can destabilize tacit values and ways of working and suggest new ways of relating—in this case—to clients’ bodies. These processes go beyond what their makers and managers anticipated. Technologies therefore actively participate in the heuristic aspect of the reflective process. The art considered here does not take place in, nor belongs to human brains only; it is performed in the conversation between human and technological actors, within practices in which these are embedded. This point is relevant too for issues raised by Sharon.

Sharon argues that instead of providing an alternative to the work of Swierstra and Verbeek, my essay serves as a supplement that fits in with the actional and relational approach already taken by these authors. I agree, but also think that the differences in emphasis that I presented—nicely summarized by Sharon as “less logos, less autos, more practice, more relationality”—are crucial. As a lens through which to study emerging networks of technologically mediated relationships, it uncovers ethical artistry that is overlooked in a focus on conscious deliberation. It enlarges the field for the study of an art of living with technologies. It also shows that the reciprocal nature of this art is better conceived of as an ‘art of becoming with’, since technological partners may not ‘live’, but their meaning and identity, too, emerges only in relationship to humans and other technologies. Taking a performative approach, I do not argue that caregivers are not reflective. I have attempted to show instead that the mode of reflexivity they demonstrate is better understood as reflection-in-action and by-action then as a process of stop-and-think, even when and where caregivers discuss their actions. Reflexivity, in so far as the human partners in the art of becoming with technologies are concerned, ranges on a scale between an actional, sensual-emotional mode and a conscious, thinking mode. However, at both ends of the scale, the main ingredients are similar: openness and experimental responsiveness to problems that challenge existing knowledge, values and procedures. At both ends, reflexivity appears as a strong reciprocal engagement with the environment, rather than as a distanced attitude. For humans, the reverse of reflexivity is not action, but routine, firm knowledge, prejudice or opinion taken for knowledge, and indifference. Technologies invite reflection even in their obtuseness. But they can seriously hinder successful reflective conversation when their design is restrictive.

Developing a relational view of the art of becoming together of humans and technologies not only emphasizes the social, material relationships in which humans are embedded; it specifically enables to include the role of practices in which humans and technologies both are embedded. Such a perspective shows caregivers as committed to trust and to respect for their clients’ dignity, and thus to having to solve the privacy issues not simply as individuals choosing these values as part of their identity, but as professionals taking their work seriously and as persons oriented to good relationships. Within care practices, as well as within other social relationships, values do not appear as personal preferences, but as transcendent to the people for whom they matter.

Another example that demonstrates the importance of considering practices, for the case at hand, is how developers of monitoring technologies did not start from scratch, choosing unfitting forms out of ignorance or bad taste. They took current care practices as their point of departure, such as the charts for registering Activities of Daily Living used to establish the ability for self care of frail clients (Glascock and Kutzik 2006). Another inspiration was traditional village life, in which people would keep track of the wellbeing of isolated neighbours by checking whether the chimney smoked in the morning. However, in the design process, registrations that used to be done on paper by caregivers in the presence of clients or by neighbours scanning the valley with their own eyes are translated into automated data collection—which may be well intended, but also causes relational trouble.

In my essay I have also suggested that a relational perspective on the value of privacy is fruitful for thinking about technomoral change in a surveillance society at large. Sharon lucidly points out the possibility of testing solutions and rules of thumb developed in this case in other cases. I think we can go further. It may be idealistic, as Sharon states, to expect companies to relate to customers on the basis of care and trust. However, consumer trust or distrust in businesses and their services does have economic impact. Also, the emphasis caregivers place on ways of ignoring and considering data ties in with already familiar ways of respecting privacy in civil relationships in public space. Goffman (1963) referred to the importance of “civil inattention” (p. 85): one doesn’t stare at others, but after acknowledging the other’s presence averts the gaze. In many current situations, technologies take us up in networks that require us to think of privacy less as a possession of individuals, and more as a quality in and of good relationships. As Boyd (2010) empathically claims with respect to privacy on social media: “we need to let go of our cultural fetishization with the individual as the unit of analysis” and develop “models that position networks, groups, and communities at the center of our discussion” (n.p.). Such positioning requires us to take another view of the self much more seriously, indeed one that is also present in Verbeek’s work: a self that doesn’t appear as an individual, regaining creative control over its personal style of life, but a permeable, responsive self. Becoming and working with technological and human others, this self attends to these relationships in artful and fitting ways, in order to live in them as well as possible, because she cares about them. Instead of being her creation, these relationships appear as what matters to her.
